# Molecular Targets in Alzheimer's Disease: From Pathogenesis to Therapeutics

**DOI:** 10.1155/2015/760758

**Published:** 2015-11-19

**Authors:** Xuan Cheng, Lu Zhang, Ya-Jun Lian

**Affiliations:** Department of Neurology, The First Affiliated Hospital of Zhengzhou University, Zhengzhou 450052, China

## Abstract

*Alzheimer's disease* (AD) is characterized by progressive cognitive decline usually beginning with impairment in the ability to form recent memories. Nonavailability of definitive therapeutic strategy urges developing pharmacological targets based on cell signaling pathways. A great revival of interest in nutraceuticals and adjuvant therapy has been put forward. Tea polyphenols for their multiple health benefits have also attracted the attention of researchers. Tea catechins showed enough potentiality to be used in future as therapeutic targets to provide neuroprotection against AD. This review attempts to present a concise map of different receptor signaling pathways associated with AD with an insight into drug designing based on the proposed signaling pathways, molecular mechanistic details of AD pathogenesis, and a scientific rationale for using tea polyphenols as proposed therapeutic agents in AD.

## 1. Introduction

Malfunction in cellular and molecular signaling is the root cause of many human diseases; and disturbances in the signaling processes and the proteins involved in the control layers are the key elements in cancer and neurodegenerative disorders [1–3]. DNA mutations often lead to inappropriate expression levels of genes encoding proteins that regulate growth, direct apoptotic machinery, repair damaged DNA, and remodel chromatin [2, 3]. Now when the signaling pathways itself is erroneous, inappropriate growth signals fail to turn on the body's cell suicide program on need and also fail to repair damaged DNA [4]. Human brain is a very complex organ and a substantial portion of the human genome is taken up with encoding brain specific signaling proteins [5]. The ion channels endow the neurons to generate action potentials which are used to signal other neurons. Imbalances between excitation and inhibition of neurons lead to abnormal patterns of neuroregulation which are responsible for epileptic seizures, deficit in attention and learning, and mood disorders. AD is a chronic neurodegenerative disorder that slowly destroys neurons leading to serious cognitive disability [1]. Epidemiologically, the disease afflicts about 5.2 million Americans with rapid escalations and the figure is expected to double by 2020. Developing countries like India and China are worst hit by this deadly disease; in 2000 India had about 3.5 million AD patients and there is an increment in the 80+ segment of the society; the numbers of Indian AD patients are increasing at an alarming rate [3].

AD is the progressive neurodegenerative disease of aging and the most common form of senile dementia. AD causes severe suffering for patients, including progressive memory loss with difficulty in performing daily activities, lack of coordination, social withdrawal, vision problems, and poor judgment. Although there are no proven modalities for curing AD, country-wise or region specific evidence based guidelines have been developed for managing AD. Conventional therapeutic regimen recommends use of major six classes of drugs which include acetylcholinesterase inhibitors (AChEI), N-methyl-D-aspartate (NMDA) receptor antagonists, monoamine oxidase inhibitors (MAOI), antioxidants, metal chelators, and anti-inflammatory drugs [1]. Apart from the first and second line of FDA recommended synthetic drugs, some of the nonpharmacologic effective preventative strategies include omega fatty acid supplementation, administration of natural antioxidants, physical activity, and cognitive engagement [2, 3].

The current scenario of drug discovery landscape has undergone a drastic change and latest pharmaceutical research aims to develop new therapeutic entities characterized by selectivity and specificity. Receptors are the proteins that reside in the plasma membrane of cells and receive signals from other molecules and are the key targets of therapeutic entities [4]. The drugs act as ligand for the receptors and can elicit pharmacologic responses in either of the two ways: the drug molecule may bind with the receptor and activate the targeted signaling pathway in the cell and the second method is the blocking mechanism where the drug acts as the null ligands that bind with the receptor but do not stimulate signaling pathways; here the drug binds with the receptor and by its blocking action prevents other ligands from binding it and activating the signaling pathway [6]. With the implementations of* in silico* and “omics” technologies and 2D and 3D quantum and docking studies now therapeutic entities are being developed targeting the enzymatic and receptor signaling pathways and drug molecules are structurally modified to achieve maximal therapeutic outcomes with minimum adverse effects [7].

## 2. Molecular Pathogenesis of Alzheimer

Molecular biology throws a significant light in studying the pathogenesis of any disease condition and AD is no exception to it. A deep insight into the understanding of the disease pathogenesis helps to develop a successful treatment regimen and realizes the existing flaws in the prevailing paradigms. Pathophysiologically, AD is complex, multifactorial, and of heterogeneous condition indicating the accumulation of amyloid cerebral plaques and neurofibrillary tangles of abnormal tau protein, presence or absence of germ line mutations, presence or absence of polymorphic susceptibility alleles, and so forth [2, 3]. The two major biochemical features related to the neuropathogenesis of AD are the neurofibrillary tangles containing phosphorylated tau protein in soluble intermediate form leading to synaptic toxicity (lack of definitive therapeutic intervention further leads to neurodegeneration) and senile plaques containing amyloid-*β*-peptide (A*β*) which is a soluble intermediate and inherently deleterious to synapses [8]. A*β* is formed after sequential cleavage of the amyloid precursor protein (APP), a transmembrane glycoprotein of undetermined function. APP can be cleaved by the proteolytic enzymes *α*-, *β*- and *γ*-secretase; A*β* is generated by successive action of the *β*- and *γ*-secretases. The *γ*-secretase, which produces the C-terminal end of the A*β*, cleaves within the transmembrane region of APP and can generate a number of isoforms of 30–51 amino acid residues in length [4, 5]. Apart from these two major proteins; oxidative stress, genetic, epigenetic, and viral hypothesis have also been put forward to explain the pathophysiology of AD [2, 3].

According to the “amyloid hypothesis,” missense mutations in the APP gene promote generation of A*β* by favoring proteolytic processing of APP by *β*- or *γ*-secretase [9]. Furthermore, APP mutations internal to the A*β* sequence heighten the self-aggregation of A*β* into amyloid fibrils [10]. Apart from these, the cloning of the presenilin (PS) proteins and AD-causing mutations in PS1 and PS2 also promote the processing of APP to form amyloidogenic A*β* [11, 12]. During AD, there is enhanced formation of A*β* which hastens the process of neuronal loss and thus it can be hypothesized that components of apoptotic machinery have a direct or indirect contribution to the complex proteolytic processing. The neuritic plaques and neurofibrillary tangles consisting of hyperphosphorylated protein tau are the major neuropathologic hallmark of AD; hence, AD is also known as “tauopathy” [2–5].

Depending on the type of secretases that cleave it, APP can undergo amyloidogenic or nonamyloidogenic processing. On being cleaved by *β*-secretase, APP via amyloidogenic pathway produces a soluble secreted form of APP (sAPP*β*) and a C-terminal fragment (*β*APP-CTF) which is further cleaved by *γ*-secretase to yield A*β* peptide and amyloid precursor protein intracellular domain (AICD) [13]. Following the nonamyloidogenic pathway, APP is first cleaved by *α*-secretase to generate the soluble secreted sAPP*α* fragment and *α*APP-CTF which is further cleaved by *γ*-secretase resulting in A*β* and AICD [14].

Multitransmembrane proteins, known as presenilins (two homologues PS1 and PS2), are catalytic components of *γ*-secretase complex having diverse biological activity and contribute to AD pathogenesis via “amyloid hypothesis.” APP and Notch (type I transmembrane cell surface receptors) are important *γ*-secretase substrates where PS plays a significant *γ*-secretase dependent role in the sequential cleavage in the processing of APP and Notch and stabilizes the *β*-catenin in Wnt signaling pathway which are *γ*-secretase independent actions. PS mutations cause impairment in the Notch signaling pathway which has significant role in neurogenesis [15–18]. The genetic inactivation of presenilins in hippocampal synapses has been shown to selectively affect the long-term potentiation caused by theta burst stimulation with the inactivation in presynapse but not the postsynapse impairing short-term plasticity and synaptic facilitation. The release of glutamate was also reduced in presynaptic terminals by processes that involve modulation of intracellular Ca^2+^ release [19]. This has been suggested to represent a general convergent mechanism leading to neurodegeneration.

From the genetic point of view, the three early onset genes, namely, the APP and the two presenilins, and the late onset gene apolipoprotein E (ApoE) significantly increase the accumulation of amyloid plaques in AD brains. ApoE, a 299-amino-acid glycoprotein with a molecular mass of 34200 Da, is a polymorphic protein. Its three isoforms (ApoE2, ApoE3, and ApoE4) in humans are all products of the same gene, which exists as three alleles (*ϵ*2, *ϵ*3, and *ϵ*4) at a single gene locus [20]. It has been demonstrated that *ε*4 allele of ApoE gene is a major genetic risk factor for late onset and sporadic AD [21, 22]. ApoE isoforms influence A*β* aggregation, modulate neurotoxicity and tau phosphorylation, play role in synaptic plasticity and neuroinflammation, elevate neurotoxicity, and retard neuroprotection [23, 24]. Therapeutic strategies modulating ApoE protein levels and its physiological and protective actions can serve as effective target in counteracting AD.

## 3. Receptor Signaling in the Pathogenesis of Alzheimer's Disease

The latest drug development pipeline focuses on pharmacological targets which include enzymes, receptors, and their different signaling pathways. Thus, a concise presentation of various AD signaling pathways which may be intra, extra, or inter, aids in drug designing approaches targeting the receptor to combat AD. From the discussions of molecular pathogenesis of AD, it is clear that presenilin/*γ*-secretase can serve as drug target [25]. Again *ε*4 allele of* ApoE* can also serve as effective therapeutic target against AD [7].

The different signaling and metabolic pathways contributing to synaptotoxicity and neurodegeneration in AD are being comprehensively focused which can serve as effective molecular targets. Amongst them are the Wnt signaling pathway, 5′-adenosine monophosphate activated protein kinase (AMPK), c-Jun-N-terminal kinases, a subfamily of mitogen activated protein kinases (MAPK), mammalian target of rapamycin (mTOR), sirtuin 1 (Sirt1, silent mating type information regulator 2 homolog 1), and peroxisome proliferator-activated receptor gamma coactivator 1-*α* (PGC-1*α*) [7, 25].

Wnt ligands interact with their receptor in the cytomembrane and subsequently activate intracellularly the signaling pathway known as the Wnt signaling pathway. In vertebrates, Wnt signaling pathway acts by programming and regulating cell proliferation, differentiation, translocation, polarization, and fate decisions [26]. Mounting evidence indicates that Wnt signaling plays an essential role in regulating the formation and function of neuronal circuits [27]. Furthermore, Wnt signaling is associated with neuron degeneration and synapse impairment, and the activation of the Wnt/*β*-catenin signaling pathway via the Wnt3a ligands (which are lipid modified signaling glycoproteins) renders protection against the toxicity of A*β* [28]. There exists a correlation between A*β*-induced neurotoxicity and diminution in *β*-catenin cytoplasmic levels. Inhibition of glycogen synthase kinase-3 beta (GSK-3*β*) renders protection against A*β*-induced damage. Coexistence of GSK-3*β* is found with neurofibrillary tangles and experimental evidences have shown that A*β* oligomers are associated with the postsynaptic region and the noncanonical Wnt signaling which modulates PSD95 and the glutamate receptors can serve the purpose of molecular target [29, 30].

AMPK, a phylogenetically conserved serine/threonine protein kinase and a metabolic sensor expressed in almost all mammalian cell types, is considered to be a novel common determinant in neurodegenerative diseases and one of the probable targets for major anti-AD drugs [31–33]. AMPK modulates intracellular ATP levels and is involved in the regulation of a number of downstream targets of several enzymatic pathways like lipolysis and glycolytic pathways [34]. Upon activation, AMPK performs a host of phosphorylations and downregulation of number of targets, namely, PGC-1*α*, which via sirt1-mediated deacetylation triggers the mitochondrial biogenesis and is involved in the direct phosphorylation of several transcription factors, and Forkhead box O3 (FOXO3) activates different transcription genes which are resistant to oxidative stress [35]. Maintenance of synaptic plasticity is maintained by enhanced mTOR activity. Evidence based research has shown that AMPK and mTOR are remarkable targets for AD [36, 37]. Opinion varies regarding the exact role of AMPK; some highlight that AMPK upon activation inhibits the phosphorylation of tau and suppresses amyloidogenesis in neurons, while other researchers have opined that AMPK phosphorylates tau and interrupts the binding of tau to microtubules. AMPK also decreases mTOR signaling, promotes lysosomal degradation of A*β*, and enhances autophagy [37, 38]. Thus, AMPK is an attractive master pharmacological target in combating AD.

JNK-mediated caspase-independent cell death plays an important role in tissue homeostasis during development. JNK signaling, a family of multifunctional signaling molecules, is activated in response to a range of stress conditions and is a potent inducer of cell death [39]. More and more evidence collected in models of AD supports the involvement of JNK signaling in AD. It has been reported that JNK could be activated by A*β* and induce the production of hyperphosphorylated Tau [40]. Moreover, inhibition of JNK with peptides prevented cell loss in model of AD [41]. Thus, more attention should be paid to not only the role of JNK in AD pathogenesis, but also its potential as a therapeutic target and biomarker.

**Figure 1:**
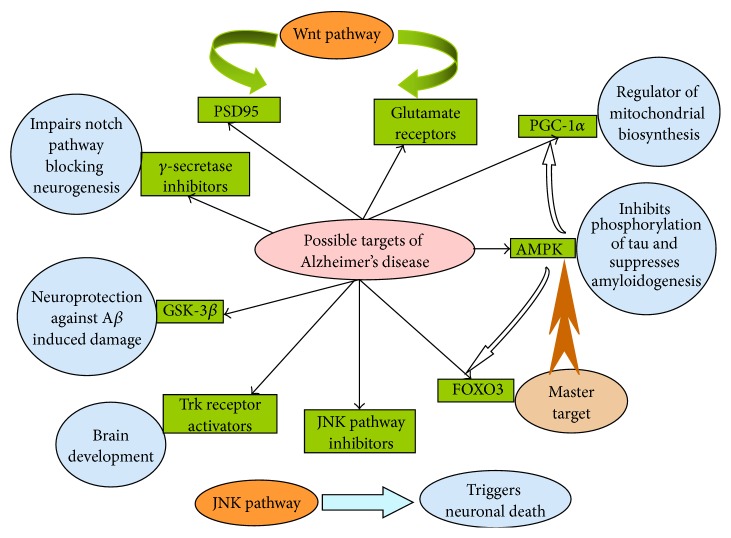
Probable therapeutic entities acting on different receptor signaling pathways of AD. AMPK: 5′-adenosine monophosphate activated protein kinase; JNK: c-Jun-N-terminal kinases; PGC-1*α*: peroxisome proliferator-activated receptor gamma coactivator 1-*α*; GSK-3*β*: glycogen synthase kinase; FOXO3: Forkhead box O3; PSD95: postsynaptic density protein 95; Trk receptor: tropomyosin receptor kinase.

Neurotrophins, namely, brain-derived neurotrophic factor (BDNF), play a critical role in neuronal survival, synaptic plasticity, and cognitive functions. BDNF is confirmed to mediate its action through various intracellular signaling pathways triggered by activation of tyrosine kinase receptor B (TrkB) [42]. All neurotrophins which activate p75 without coactivation of the concerned Trk receptors induce apoptosis of the hippocampal neurons [43]. From the pathogenic point of view, the neuronal death is triggered if Trk is not activated, and A*β* activates p75 through neurotrophines. Therapeutic agents that activate Trk may be useful in counteracting the situation [44]. The diagrammatic presentation of different receptor signaling pathways of AD is presented in Figure 1.

## 4. Adjuvant Therapy as Probable Therapeutic Entities in Alzheimer

Considering the available therapeutic regimen with synthetic drugs, the major six classes of drugs recommended in the treatment of AD include AChEI, NMDA receptor antagonists, antioxidants, MAOI, and anti-inflammatory agents. AChEI are the first-line drugs in treating mild to moderate AD. FDA approved five prescription drugs currently in use to control symptoms of AD amongst which donepezil, galantamine, rivastigmine, and tacrine are the AChEI and memantine comes under NMDA receptor antagonists. However, tacrine had to be withdrawn due to severe hepatotoxicity and none of the available drugs are free from the side effects of gastrointestinal disturbances, nausea, vomiting, headache, and so forth [1–3].

Reactive oxygen and nitrogen species (ROS and RNS, resp.) leading to oxidative and nitrosative stress cause destruction of brain macromolecules leading to neurodegeneration. Clinical and epidemiological research evidences suggest that intake of polyphenols (either flavonoids or nonflavonoids) from natural sources can reduce the risk of AD [45–47]. Flavonoid mediated neuroprotection is possible only when they are able to cross the blood-brain barrier (BBB). Epigallocatechin gallate (EGCG), a polar polyphenol, and methylated flavonoids are found to cross the BBB successfully after gastric administration [48].

Tea, the most popular beverage, has attracted attention as an adjuvant therapy in treating many disease conditions in a nonpharmacologic manner. Health benefits of tea are highly correlated with total polyphenolic contents (TPC). Currently there has been a great resurgence of interest in adjuvant therapy in combating different chronic and neurodegenerative disorders. The remarkable antioxidant potentials of tea catechins, namely, epicatechin (EC), epicatechin gallate (ECG), epigallocatechin (EGC), and EGCG, have already attracted research attention [46, 47]. This section of the paper attempts to highlight the probabilities of using tea polyphenols as potential therapeutic agents for AD. Antioxidant potencies of tea catechins follow the order of EGCG>ECG>EGC>EC [49, 50]. Catechins by ultrarapid electron transfer to ROS induced radical sites on DNA can give effective protection against ROS induced damages [51]. EGCG and chelates with metal ions prevent the suppression of the translation of APP mRNA. EGCG modulates cell apoptosis and protects against oxidative stress [48]. It inhibits caspase-3 and activates the PI3K/Akt pathway, restores the activity of protein kinase C (PKC), and promotes cell survival. EGCG inhibits the expression of proapoptotic genes* Bax* and* Bad* and induces antiapoptotic genes, namely, Bcl-2, Bcl-W, and Bcl-X [52]. It has already been mentioned that anti-inflammatory agents find use in the prophylaxis of AD. Transcription factor nuclear factor-kappa B triggered in response to oxidative stress plays a key role in inflammation. The galloyl and hydroxyl moieties at the 3′ position on EGCG contribute to its strong anti-inflammatory properties. However, biological activities of EGCG are highly concentration dependent. EGCG activates PKC and enhances the release of nonamyloidogenic soluble precursor through a PKC dependent pathway confirming the role of PKC in the neuroprotective role of EGCG. EGCG provides neuroprotection and combats cognitive impairment by promoting expression of TrkA phosphorylation, reduces JNK activation, and inhibits expression of cleaved caspase-3, thus counteracting the formation of A*β* and APP in the hippocampal regions [53–55].

## 5. Conclusion

AD being a fatal, progressive neurodegenerative disorder, with multiple complications and cognitive impairments development of new therapeutic entities with minimal side effects, is of utmost importance. Available marketed first-line synthetic drugs show one to more side effects. Current research trend aims to develop pharmacological targets based on their enzymatic and receptor signaling pathways. Nutraceuticals and adjuvant therapy have also attracted attention of researchers. Tea is a popular beverage and the tea catechins, namely EGCG (in both green and black tea), theaflavins, and thearubigins, the black tea polyphenols, showed enough potentiality to provide neuroprotection against AD, based on the upregulation or downregulation of the signaling pathways influencing the disease pathogenesis. However, further extensive research is warranted in this regard. On establishment of the effectivity of tea polyphenols in combating AD, it will undoubtedly promote the growth of tea industry with research expansions via academy-industry collaborations.
